# Evaluation of Creatine Kinase, Lactate Dehydrogenase, and Amylase Concentrations in Umbilical Blood of Preterm Infants after Long-Term Tocolysis

**DOI:** 10.1155/2014/278379

**Published:** 2014-02-13

**Authors:** Yoshiyuki Nakajima, Naoki Masaoka

**Affiliations:** Department of Obstetrics and Gynecology, Tokyo Women's Medical University Yachiyo Medical Center, 477-96 Owadashinden, Chiba, Yachiyo, 276-8524, Japan

## Abstract

Creatine kinase (CK), lactate dehydrogenase (LDH), and amylase levels of preterm infants following long-term tocolysis in pregnant women are limited. The objective of this study was to determine if the tocolytic therapy affects CK, LDH, and amylase levels in the umbilical blood. This study included 215 preterm infants born to women treated with and without ritodrine hydrochloride. CK, LDH, and amylase levels in the umbilical blood at delivery were determined. Infants were divided according to the ritodrine tocolysis, as follows: Group A (n = 91), not exposed to ritodrine; Group B (n = 44), IV ritodrine for <1 week; Group C (n = 80), IV ritodrine for ≥1 week. The CK concentration in cord blood of Group C (198.8 ± 14.2 IU/L) was significantly higher in comparison with Group A (155.0 ± 7.3 IU/L, P < 0.05). There was no significant difference in LDH and amylase levels in the three groups. The CK significantly correlated with gestational age (r = 0.42, P < 0.01) and birth weight (r = 0.38, P < 0.01). LDH and amylase levels did not change with gestational age nor birth weight. In conclusion, long-term ritodrine tocolysis leads to increased umbilical blood CK level.

## 1. Introduction

Recently, the incidence of preterm birth has increased, despite rapid advances in maternal-fetal medicine. The beta-adrenergic agent ritodrine hydrochloride is commonly used for the treatment of preterm labor in Japan. Numerous adverse effects have been reported during tocolysis with such drugs, including cardiac, pulmonary, and metabolic effects [1]. Asymptomatic increases in blood creatine kinase (CK) and amylase have been reported in pregnant women treated with tocolytic agents [2–5]. Although ritodrine hydrochloride crosses the placenta freely [6], the data concerning escape enzyme, such as CK, lactate dehydrogenase (LDH), and amylase levels of preterm infants following long-term ritodrine tocolysis in pregnant women are limited.

We hypothesized that ritodrine hydrochloride would affect the preterm fetuses, thereby adversely affecting escape enzyme levels. Therefore, the aim of this study is to determine if the tocolytic therapy affects CK, LDH, and amylase levels in the umbilical blood of preterm infants.

## 2. Methods

To examine the clinical significance of the relationship between ritodrine hydrochloride and escape enzymes in the preterm infants, we reviewed all patients who were delivered at less than 36 weeks of gestation with preterm birth in the Perinatal Center of Social Insurance Funabashi Central Hospital between July 1, 2005, and June 30, 2008. Informed consent was obtained from all subjects and all study protocols were approved by the institutional review board before the start of this study. Subjects were included preterm labor, premature rupture of membranes (PROM), and cervical insufficiency. Preterm labor was defined as demonstrated progressive dilation of the cervix with uterine contractions before 36 weeks of gestation. PROM was documented by sterile speculum examination, confirming the pooling of fluid in the posterior vaginal fornix, an alkaline pH, ferning, and direct visualization of fluid leakage from the cervical canal. Cervical insufficiency was defined as premature effacement or dilatation of the cervix in the absence of labor or ultrasonographic evidence of cervical shortening less than 25 mm or funneling. Subjects were excluded if at the time of admission they had intrauterine growth restriction (IUGR), pregnancy-induced hypertension (PIH), multiple pregnancy, abruptio placenta, chromosomal abnormality, or stillbirth. Moreover, pregnant women who did not receive glucocorticosteroids for accelerating fetal organ maturation and decreasing the risk of respiratory distress syndrome (RDS) and intraventricular hemorrhage (IVH) were eligible for this study. Gestational age was estimated based on menstrual dates and an ultrasound examination performed before 20 weeks of gestation. PIH was diagnosed where there was systolic blood pressure higher than 140 mm Hg and/or diastolic blood pressure higher than 90 mm Hg after 20 gestational weeks. IUGR was defined as estimated weight less than the 10th percentile of the Japanese standard [7]. Clinical findings and maternal and neonatal outcomes were recorded from medical records, retrospectively reviewed, and analyzed. Clinical data included maternal age at birth, parity, maternal medical history, family history, antepartum complications, antepartum treatment, fetal heart rate (FHR) monitoring, and laboratory findings. The major obstetric outcomes were maternal morbidity and mortality, interval to delivery, and indications for and mode of delivery. The main reasons for preterm delivery were advanced labor and nonreassuring FHR pattern. Nonreassuring FHR patterns were considered present when one of the following FHR patterns was detected: recurrent late deceleration, recurrent severe variable deceleration, prolonged deceleration, or loss of variability [8].

Ritodrine hydrochloride and magnesium sulfate were administered as tocolytic agents for preterm labor and cervical insufficiency [1]. The initial dose of ritodrine hydrochloride was 50 *μ*g/min, which was increased by 50 *μ*g/min every 10–20 min until uterine contractions ceased. When control of uterine contractions could not be achieved even with administration of 200 *μ*g/min of ritodrine, magnesium sulfate was added to the regimen. The method of administration was based on that described by Zuspan and Ward [9], with intravenous injection of 4 g of magnesium sulfate over 30 min followed by 1-2 g/h with close monitoring of serum magnesium concentrations. Once PROM was diagnosed, the patients were not treated with tocolytic agents. Delivery was initiated at the onset of active labor, clinical chorioamnionitis, or nonreassuring fetal status. Cesarean sections were performed for those patients who had previously undergone cesarean section as well as those in whom nonreassuring fetal status or fetal malpresentation was identified.

On admission and every week thereafter, maternal blood tests including serum CK, LDH, and amylase were performed. Umbilical venous blood was obtained from all infants at delivery. The serum was immediately analyzed for CK, LDH, and amylase, using an automatic analyzer with analysis at 37°C (Hitachi 7170, Tokyo, Japan).

Neonatal data dealing with gestational age, birth weight, 1 min and 5 min Apgar scores, gender, umbilical cord pH, Kaup index, and neonatal complications (RDS, IVH, patent ductus arteriosus (PDA) necrotizing enterocolitis, and retinopathy) were recorded.

Patients were divided into three groups according to the use of ritodrine hydrochloride during the antepartum period, as follows: Group A, preterm infants who were not exposed to ritodrine hydrochloride in utero, Group B, preterm infants after short-term tocolysis, which means the mothers of these infants were given intravenous infusion of ritodrine hydrochloride for <1 week; Group C, preterm infants after long-term tocolysis by intravenous infusion of ritodrine hydrochloride for ≥1 week. Moreover, two subsets of each group were further evaluated: those with no exposure to magnesium sulfate in utero (Groups A1, B1, and C1) and those after tocolysis by intravenous infusion of magnesium sulfate (Groups A2, B2, and C2).

Results are expressed as mean ± standard error of mean (SEM). Clinical characteristics were compared among the groups using a Kruskal-Wallis test, followed by the Mann-Whitney *U *test for continuous variables, as well as the chi-square or Fisher's exact test to compare proportions. The relationship between escape enzymes and gestational age or birth weight were studied by regression analysis. Correlations between levels of escape enzymes and gestational age or birth weight was calculated for data from Groups A, B, and C and all infants were combined into a single group. The statistical package used was SPSS, version 21 (SPSS, Chicago, IL). Values of *P* < 0.05 were considered statistically significant.

## 3. Results

A total of 215 cases were enrolled in this study, with 91 cases in Group A, 44 cases in Group B, and 80 cases in Group C. Clinical background data for the four groups are compared in Table 1.

No significant differences were identified between all groups in terms of maternal age, gravidity, parity, body mass index at delivery, gestational age at delivery, birth weight, sex male, Apgar score at 1 and 5 min, umbilical arterial pH, Kaup index, and placental weight. The frequency of administration of magnesium sulfate of Group C was significantly higher than that of Group A (*P* < 0.01) and Group B (*P* < 0.05). The rate of cesarean delivery of Group B was significantly lower than that of Group A (*P* < 0.01) and Group C (*P* < 0.01). Moreover, the CK concentration in maternal blood of Group C was significantly higher in comparison with Group B. There was no significant difference in LDH and amylase levels in the three groups. No pregnant women showed any signs of rhabdomyolysis and pancreatitis.

**Figure 1:**
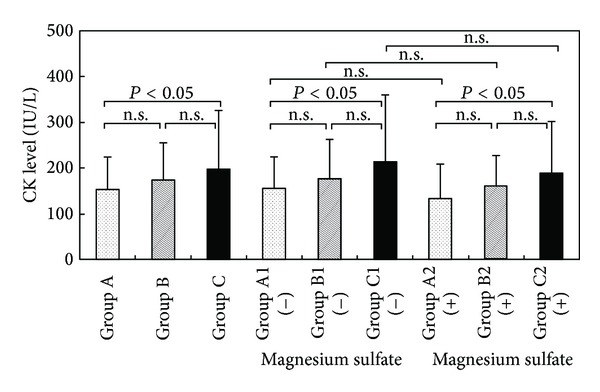
CK levels in umbilical blood after tocolysis. *P < 0.05 (compared with control group).

Figure 1 shows a comparison of CK levels in the umbilical blood of preterm infants. CK levels were 155.0 ± 7.3 IU/L in Group A, 173.7 ± 12.4 IU/L in Group B, and 198.8 ± 14.2 IU/L in Group C. The CK concentration in cord blood of Group C was significantly higher in comparison with Group A (*P* < 0.05). CK concentrations were significantly higher in Groups C1 and C2 than in Groups A1 and A2, respectively (*P* < 0.05). No significant differences in CK levels were seen between Groups A1 and A2, Groups B1 and B2, or Groups C1 and C2.

**Figure 2:**
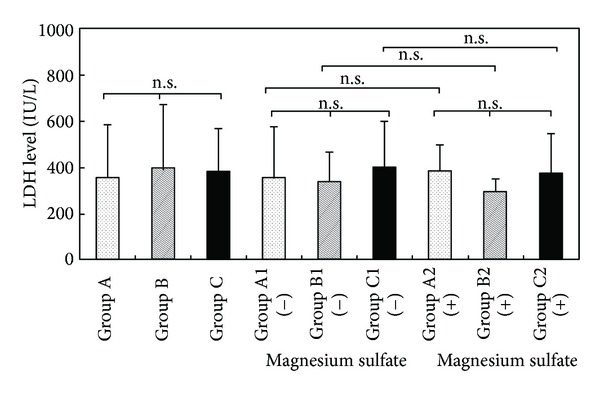
LDH levels in umbilical blood after tocolysis.

Figure 2 shows a comparison of LDH levels in the umbilical blood of preterm infants. LDH levels were 365.7 ± 26.4 IU/L in Group A, 391.8 ± 65.0 IU/L in Group B, and 377.6 ± 20.6 IU/L in Group C. There was no significant difference in LDH levels in the three groups.

**Figure 3:**
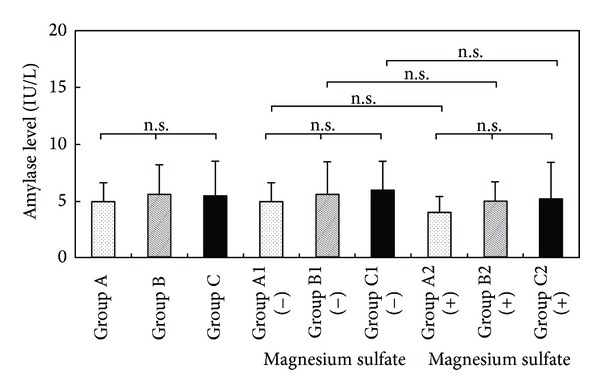
Amylase levels in umbilical blood after tocolysis.

Figure 3 shows a comparison of amylase levels in the umbilical blood of preterm infants. Amylase levels were 4.9 ± 0.2 IU/L in Group A, 5.6 ± 0.6 IU/L in Group B, and 5.4 ± 0.4 IU/L in Group C. There was no significant difference in amylase levels in the three groups.

**Figure 4:**
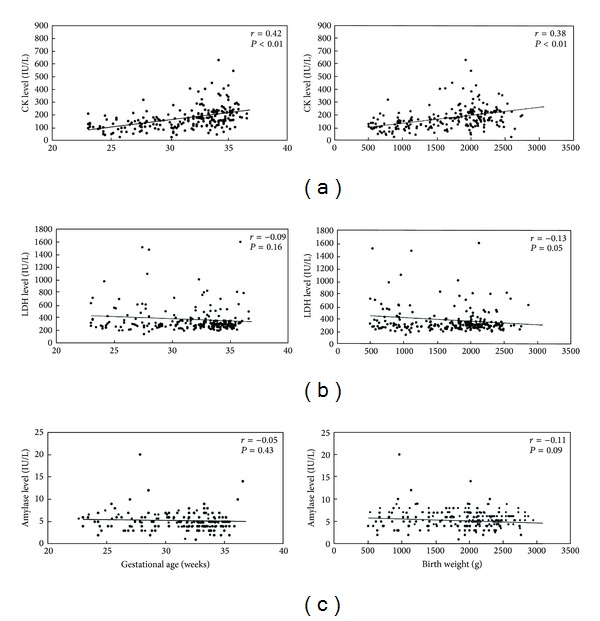
Correlation between CK, LDH and amylase levels and gestational age and birth weight in total patients.

Figure 4 shows the relationship between CK (Figure 4(a)), LDH (Figure 4(b)), and amylase (Figure 4(c)) levels and gestational age or birth weight. In all infants, positive linear correlations were observed between CK level and gestational age (*r* = 0.42, *P* < 0.01) and birth weight (*r* = 0.38, *P* < 0.01). LDH and amylase levels did not change with gestational age (LDH, *r* = 0.09; amylase, *r* = 0.05) nor birth weight (LDH, *r* = 0.13; amylase, *r* = 0.11).

**Figure 5:**
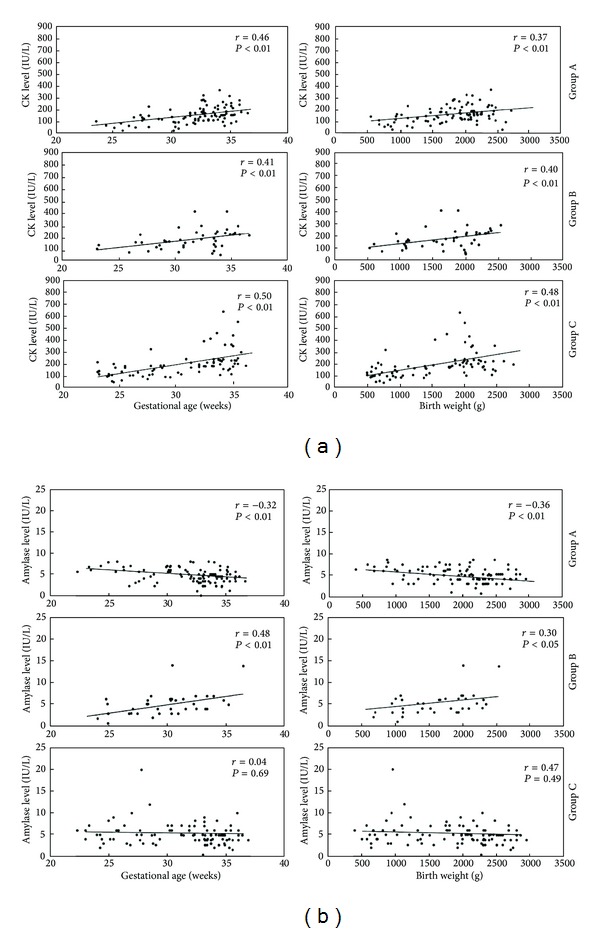
Correlation between CK (a) and amylase (b) levels and gestational age and birth weight in the three groups. (a) Correlation between CK levels and gestational age and birth weight in the three groups. (b) Correlation between amylase levels and gestational age and birth weight.

As in Figure 1, the CK concentration was significantly higher in infants after long-term tocolysis in comparison with the control group, suggesting that the difference in skeletal muscle mass. It was confirmed by the correlation between CK and gestational age (Group A, *r* = 0.46, *P* < 0.01; Group B, *r* = 0.41, *P* < 0.01; Group C, *r* = 0.50, *P* < 0.01) or birth weight (Group A, *r* = 0.37, *P* < 0.01; Group B, *r* = 0.40, *P* < 0.01; Group C, *r* = 0.48, *P* < 0.01) in each group (Figure 5(a)).

LDH did not change with gestational age (Group A, *r* = −0.07; Group B, *r* = −0.10; Group C, *r* = −0.13) nor with birth weight (Group A, *r* = −0.11; Group B, *r* = −0.14; Group C, *r* = −0.17) (data not shown).

In Group A, negative linear correlations were observed between amylase level and gestational age (*r* = −0.32, *P* < 0.01) or birth weight (*r* = −0.36, *P* < 0.01). In Group B, positive linear correlations were observed between amylase level and gestational age (*r* = 0.48, *P* < 0.01) or birth weight (*r* = 0.30, *P* < 0.05). In Group C, amylase did not change with gestational age (*r* = 0.04) nor with birth weight (*r* = 0.07) (Figure 5(b)).

## 4. Discussion

The purpose of this study was to investigate relationships between ritodrine hydrochloride and escape enzymes in the preterm infants.

There is some controversy regarding the optimum duration of tocolysis. In Japan, treatment is expected to prolong pregnancy as much as possible by suppressing uterine contractions enough to delay delivery but, in the US and Canada, it is used to maintain pregnancy by substantial and rapid inhibition of concentrations for a period long enough to enable maturation of fetal lung to be promoted with corticosteroids [10]. Takagi et al. reported that administration of ritodrine hydrochloride occurred in 86% patients in Japan participating in the Multicenter Premature Labor Study Group and IV ritodrine hydrochloride was considered safe and effective for prolonging gestation in patients of preterm labor [11].

CK and LDH are nonspecific markers of tissue and muscle injury. Preliminary studies suggest that fetal or umbilical cord blood enzymes at delivery reflect the effects of labor and drugs. Adamcová et al. reported that CK level correlated significantly with gestational age or birth weight [12]. Weiner et al. reported LDH rose with advancing gestation [13]. Nava et al. reported that amylase levels did not change with gestational age [14]. In our study, CK levels in patients with and without ritodrine administration, in agreement with previous studies, supported a relationship between CK levels and gestational age or birth weight. LDH levels in all groups, in agreement with these studies, tended to support the relationship between LDH levels and gestational age or birth weight, but no significant relationship was identified in the present study. Amylase levels in total decreased with advancing gestation or birth weight, a finding that might be explained by the sparse pancreatic function in the fetus. These changes found in CK and amylase levels were not pathological but physiological.

Although ritodrine hydrochloride is generally considered fairly safe [1], no reports have addressed the relationship between ritodrine hydrochloride and escape enzymes in the umbilical blood of preterm infants. We thus sought to determine whether differences exist in umbilical cord enzymes concentrations when subdivided according to the presence or absence of ritodrine tocolysis. The mechanisms of onset for hypercreatine kinasemia vary with the cause [1]. One hypothesis is that CK levels could reflect both direct and indirect cellular damage. First, supporting the possibility of direct muscular damage, Weidinger et al. reported the necrosis of fetal myocardial cells treated with beta-agonists *in vitro* [15]. Second, indirect damage may be attributable to hypokalemia, a side effect of beta-agonists. During muscular contraction, interstitial arteries are dilated by potassium, so a lack of this ion following administration of beta-agonists may cause transient muscular ischemia, with consequent indirect muscular damage [16].

LDH is virtually ubiquitous in the body, and several tissues, such as heart, liver, skeletal muscle, and kidney, show a tissue-serum ratio greater than 1000, and hence raised values are of nonspecific clinical significance. The mechanisms leading to extracellular release of LDH are still poorly understood. Pathological events include ischemia, shock, toxic, and inflammatory conditions and mechanical and physical destruction of tissue [17]. Perkins et al. reported cord blood LDH activities to be approximately twice those of adult values [18]. This confirms results from Freer et al. [19], who also found that the LDH isoenzyme 5 contributed to a large portion of this increase. The mechanisms underlying elevated LDH levels in association with ritodrine hydrochloride are unclear; however, myocardial damage may be due to ritodrine hydrochloride [1]. In addition, liver impairment may be due to overloading of hepatic function resulting from metabolic changes induced by ritodrine hydrochloride [20].

Ritodrine hydrochloride can induce hyperamylasemia in pregnant women [21]. Takahashi et al. reported that the hypothesis that beta-adrenergic agents induced hyperamylasemia happens through the stimulation of beta-adrenoceptors of the salivary glands. In their study, clinical doses of ritodrine hydrochloride induced hypersecretion of salivary-type amylase in approximately one-third of pregnant women. Moreover, peak serum amylase activity in the maternal blood was seen on day 1, began to decrease on day 2, and returned to initial values within 14 days, despite continuous ritodrine infusion. The number of beta-adrenoceptors has been shown to decrease in salivary glands of rats treated with beta-adrenergic agent and demonstrates that the down-regulation of beta-adrenoceptors in the tissue can occur [22]. Takahashi et al. suggested that desensitization to ritodrine hydrochloride might occur after prolonged use [21]. An increase in this enzyme in cord blood has not been established and is particularly interesting. In the present study, there was no significant difference in amylase levels in the three groups.

Moreover, in control group, negative linear correlations were observed between amylase level and gestational age or birth weight. However, in short-term ritodrine tocolysis, positive linear correlations were observed between amylase level and gestational age or birth weight. As ritodrine hydrochloride freely diffuses across the placenta, this positive linear correlation means that the increased cord amylase level might be due to a direct effect of the ritodrine hydrochloride in infants with higher ritodrine concentration in short-term ritodrine tocolysis. However, in long-term ritodrine tocolysis group, no correlation was found between amylase levels and gestational age or birth weight. There was an inverse relationship between short-term tocolysis and long-term tocolysis. Although the reason for this is unknown, it may be related to desensitization to ritodrine hydrochloride that was suggested by Takahashi et al. [21]. Concerning desensitization, Auman et al. reported the disparity between regulatory responses in the developing heart compared with other organs [23]. Neonates and adults show different responses to long-term **β**-adrenergic stimulation, with an absence of desensitization in the neonatal heart. Other immature organs are capable of homologous desensitization in response to **β**-agonists.

The objective of this retrospective analysis was to clarify the variations in escape enzyme levels in umbilical cord blood when ritodrine hydrochloride with or without magnesium sulfate was administered as tocolytic therapy. We found that the CK concentration was significantly higher with long-term ritodrine tocolysis than with no ritodrine administration. In addition, significant differences were seen between all groups in the frequency of concomitant administration of magnesium sulfate. One possibility is that magnesium sulfate may have additive effects on CK levels over those of ritodrine hydrochloride. Subsets with and without magnesium sulfate were thus further evaluated, and we found that CK concentrations in those with long-term ritodrine tocolysis were significantly higher than in those without ritodrine administration.

Our results point to another area for future investigation: the potential for differences in mode of delivery to affect fetal responses to tocolytic therapy. Significant differences in the rate of cesarean delivery were apparent among the three groups. Mode of delivery may thus contribute to the levels of escape enzymes in the umbilical blood of preterm infants. Some controversy exists regarding the effects of mode of delivery on escape enzyme levels in umbilical cord blood. Leiserowitz et al. reported that pregnant women having a vaginal delivery show significantly elevated CK and CK-MB levels [24]. Conversely, Liras et al. reported that significant differences were found in CK-MB activity, but not in total CK, between cord blood samples from vaginal births and those from cesareans [25]. In the present study, although no significant difference was identified between the control and long-term ritodrine tocolysis groups in the rate of cesarean delivery, CK concentrations in cord blood were significantly higher in the long-term ritodrine tocolysis group than in the control group.

Although the transfer of CK through the placenta cannot be excluded, because its molecular weight is 82 kDa, our data support the idea that the transfer of CK across the placenta is limited. A major limitation in this study was the relatively small number of patients. Further investigation with a larger number of patients is thus warranted.

## 5. Conclusions

Long-term tocolysis should be taken into account in the clinical interpretation of CK levels in umbilical blood. Further studies are necessary to confirm the relationship between ritodrine hydrochloride and escape enzymes in the umbilical blood of preterm infants. Further prospective study is needed to confirm findings.

## Figures and Tables

**Table 1:** Clinical backgrounds of the three groups.

	Group A (*n* = 91)	Group B (*n* = 44)	Group C (*n* = 80)	*P* value
Maternal age	31.3 ± 0.5	31.6 ± 0.8	32.1 ± 0.6	N.S.
Gravidity	1.6 ± 0.2	1.4 ± 0.4	1.2 ± 0.3	N.S.
Parity	0.9 ± 0.1	1.0 ± 0.1	0.7 ± 0.1	N.S.
Body mass index at delivery	24.4 ± 0.5	23.4 ± 0.7	23.3 ± 0.7	N.S.
Magnesium sulfate (%)	5.8**	17*	58.1^∗∗,∗^	<0.01** <0.05*
Gestational age (weeks)	31.3 ± 0.5	31.6 ± 0.8	32.1 ± 0.6	N.S.
Birth weight (g)	1776.1 ± 59.4	1718.7 ± 88.0	1606.6 ± 65.1	N.S.
Cesarean birth (%)	67.4	37**	68.8**	<0.01**
Sex male (%)	55.6	54.6	62.7	N.S.
1 min Apgar score	7.1 ± 0.2	7.3 ± 0.4	6.6 ± 0.2	N.S.
5 min Apgar score	8.5 ± 0.1	8.7 ± 0.2	8.2 ± 0.2	N.S.
Umbilical arterial pH	7.29 ± 0.02	7.34 ± 0.04	7.32 ± 0.02	N.S.
Kaup index	10.3 ± 0.2	10.1 ± 0.3	10.0 ± 0.2	N.S.
Placental weight (g)	448.2 ± 12.3	438.4 ± 17.9	442.4 ± 13.5	N.S.
Maternal blood CK (IU/L)	55.5 ± 40.9	49.4 ± 45.4*	158.0 ± 501.7*	<0.05*
Maternal blood LDH (IU/L)	170.3 ± 40.8	169.5 ± 30.4	181.1 ± 67.7	N.S.
Maternal blood amylase (IU/L)	79.2 ± 26.2	72.5 ± 26.5	75.4 ± 23.9	N.S.

N.S.: not significant; *P < 0.05; **P < 0.01.

## Abbreviations

CK: Creatine kinase
LDH: Lactate dehydrogenase
IUGR: Intrauterine growth restriction
PIH: Pregnancy-induced hypertension
RDS: Respiratory distress syndrome
IVH: Intraventricular hemorrhage
FHR: Fetal heart rate
PDA: Patent ductus arteriosus
SEM: Standard error of mean.
